# Selective Serotonin Reuptake Inhibitors Potentiate the Rapid Antidepressant-Like Effects of Serotonin_4_ Receptor Agonists in the Rat

**DOI:** 10.1371/journal.pone.0009253

**Published:** 2010-02-16

**Authors:** Guillaume Lucas, Jenny Du, Thomas Romeas, Ouissame Mnie-Filali, Nasser Haddjeri, Graciela Piñeyro, Guy Debonnel

**Affiliations:** 1 Department of Psychiatry, Centre de Recherche Fernand Seguin, Université de Montréal, Montréal, Québec, Canada; 2 EAC CNRS 3006, ISPB Université de Lyon 1, Faculty of Pharmacy, Lyon, France; 3 Department of Psychiatry, Bâtiment de Recherche et de Formation, Université McGill, Montréal, Québec, Canada; Tokyo Medical and Dental University, Japan

## Abstract

**Background:**

We have recently reported that serotonin_4_ (5-HT_4_) receptor agonists have a promising potential as fast-acting antidepressants. Here, we assess the extent to which this property may be optimized by the concomitant use of conventional antidepressants.

**Methodology/Principal Findings:**

We found that, in acute conditions, the 5-HT_4_ agonist prucalopride was able to counteract the inhibitory effect of the selective serotonin reuptake inhibitors (SSRI) fluvoxamine and citalopram on 5-HT neuron impulse flow, in Dorsal Raphé Nucleus (DRN) cells selected for their high (>1.8 Hz) basal discharge. The co-administration of both prucalopride and RS 67333 with citalopram for 3 days elicited an enhancement of DRN 5-HT neuron average firing rate, very similar to what was observed with either 5-HT_4_ agonist alone. At the postsynaptic level, this translated into the manifestation of a tonus on hippocampal postsynaptic 5-HT_1A_ receptors, that was two to three times stronger when the 5-HT_4_ agonist was combined with citalopram. Similarly, co-administration of citalopram synergistically potentiated the enhancing effect of RS 67333 on CREB protein phosphorylation within the hippocampus. Finally, in the Forced Swimming Test, the combination of RS 67333 with various SSRIs (fluvoxamine, citalopram and fluoxetine) was more effective to reduce time of immobility than the separate administration of each compound.

**Conclusions/Significance:**

These findings strongly suggest that the adjunction of an SSRI to a 5-HT_4_ agonist may help to optimize the fast-acting antidepressant efficacy of the latter.

## Introduction

The recourse to bi-, tri- or even multi-therapy is not uncommon in current clinical practice. Obviously, one of the most illustrative examples resides in how “the tri-therapy” has become popular to treat AIDS patients. In the psychiatric field, though, the use of multi-therapy as a first line treatment for a single affection is far from being a systematic option. It is true that many “atypical” antipsychotics have been developed to bind several sites within the brain. However, as pinpointed by Kapur and Remington in 1996 [1], these molecules have, by definition, a fixed ratio of affinities for their different targets, and, unlike a cocktail of distinct active principles, do not permit to modulate each of them in an independent manner. To define such a strategy as a “multi-therapy” would therefore appear abusive, if not inappropriate. Similarly, none of the antidepressant treatments that have been routinely used so far are based on a bi-(or multi-)therapy concept. Interestingly, recent *in vivo* studies, using a wide dose-range, suggest that even the “mixed” (i.e. with a fixed ratio of affinities) serotonin (5-HT) and norepinephrine (NE) reuptake blockers venlafaxine and duloxetine act mostly as selective serotonin reuptake inhibitors (SSRIs) after systemic administration [2]. Although the adjunction of atypical antipsychotics to antidepressants may have a therapeutic interest in some depressed patients [3]-[4], this combination remains a second-line solution, used only after the more “classical” molecules have revealed ineffective [4]–[5]. In addition, the rationale for this strategy, as well as the underlying biological mechanism(s), remains to be determined [4]–[5].

Yet, a dual approach, involving distinct and independent actions within the brain, might reveal of high interest in the context of depression. Indeed, the main challenge posed by current treatments resides in their delayed onset of action, the therapeutic improvement being observable only after 4 to 8 weeks of continuous administration [6]–[8]. According to the “serotonergic theory” of depression, this delay is related to the presence of inhibitory 5-HT_1A_ autoreceptors on 5-HT cell bodies [9]–[10]. These autoreceptors actually trigger a strong inhibition of 5-HT neuron firing rate, counteracting almost totally the passive elevation of 5-HT extracellular levels that the above cited molecules induce by blocking the inactivation (catabolism or re-uptake) of the transmitter [9]–[10]. It is believed that the latency of classical antidepressants corresponds precisely to the period required for 5-HT_1A_ autoreceptors to become desensitized [7], [9]–[10]. Based on these considerations, it has been proposed that one possibility to reduce the delayed onset of antidepressant action would reside in a direct activation of 5-HT neuron firing rate, bypassing the presynaptic 5-HT_1A_ control [6], [11]. In this context, we have recently reported that 5-HT_4_ receptor agonists can induce such an activation [12]–[13], and that they actually may constitute a novel, fast-acting class of antidepressants [14]. However, the question whether it would be possible to combine a direct enhancement of 5-HT neuronal impulse flow with the passive augmentation of 5-HT levels produced by conventional antidepressants still remains unanswered. Indeed, it appears reasonable to expect that such a double action should produce an optimal increase of the central 5-HT transmission. Given the apparent importance of this parameter in antidepressant efficacy [15], this might constitute a significant breakthrough for patients, as more than 60% of them fail to respond, or only partially do, to classical treatments [16].

The present study was conducted to assess the ability of various SSRIs to augment the effect of the selective 5-HT_4_ agonists prucalopride and RS 67333, in several experimental paradigms considered to reflect an antidepressant action. Thus, the chosen protocols were focused on the study of central 5-HT transmission, at both the pre- and postsynaptic levels, on the phosphorylation of the cAMP-response element binding (CREB) protein within the hippocampus, as well as on the response to several combined treatments in the forced swimming test (FST) [14].

## Results

### Effect of acute co-administrations of an SSRI and of the selective 5-HT_4_ agonist prucalopride on DRN 5-HT neuronal firing rate

**Figure 1:**
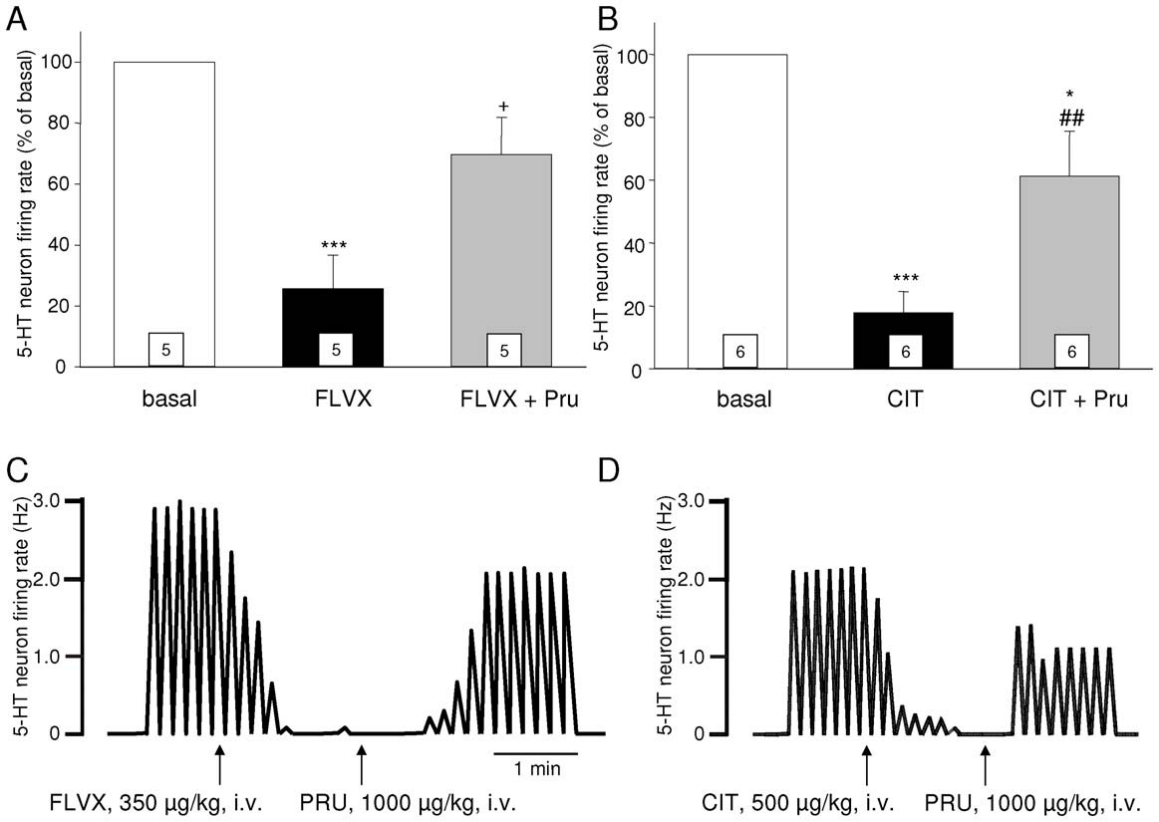
Effect of an acute administration of the 5-HT_4_ agonist prucalopride (1000 μg/kg, i.v.) on the inhibition of DRN 5-HT neuron firing rate induced by the SSRIs fluvoxamine and citalopram. **A** and **B**: Summary of the results found with fluvoxamine (FLVX; 350 µg/kg, i.v.) and citalopram (CIT; 500 µg/kg, i.v.), respectively. Bar histograms represent the mean (± S.E.M.) percentage effect, calculated for each neuron with respect to its basal firing rate (i.e., 100% level), and the value at the bottom of each column indicates the number of neurons tested. Only one single neuron was recorded per animal. * p<0.05 and *** p<0.001 vs basal, + p<0.05 vs fluvoxamine, ## p<0.01 vs citalopram, Tukey's test. Panels **C** and **D** show individual examples of integrated firing rate histograms, in the cases of a fluvoxamine and a citalopram administration, respectively.

In all the recorded cells, the intravenous injection of either fluvoxamine (350 µg/kg, n = 10) or citalopram (500 µg/kg, n = 11) strongly reduced 5-HT neuron impulse flow. An illustration of this effect is shown in Figs. 1A and 1B, showing a decrease to 26% and 18% of basal (pre-drug) values in the presence of fluvoxamine and citalopram, respectively. Similar results, thought to reflect the increased stimulation of somatodendritic 5-HT_1A_ autoreceptors that follows 5-HT reuptake blockade [17]–[18], have already been reported when using these SSRIs [18]–[20]. At difference with what was observed with SSRIs, two distinct populations of 5-HT neurons were found regarding their response to subsequent injection of the 5-HT_4_ agonist prucalopride (1000 µg/kg, i.v.). Indeed, in cells specifically chosen for their low (≤1 Hz) basal firing rate, the 5-HT_4_ agonist was unable to affect the inhibitory action of either compound (n = 5 each, not shown). In contrast, when 5-HT neurons were selected on the basis of a high (≥2 Hz) basal discharge, prucalopride counteracted the effect of both fluvoxamine (n = 5) and citalopram (n = 6) (Figs. 1C and 1D). The observation of such a dichotomy is in well agreement with our previous study, describing the existence of a sub-population of DRN 5-HT neurons “responding” to 5-HT_4_ receptor stimulation (47%), the other ones remaining insensitive and having been dubbed as “non-responders” [12]. In addition, the mean basal firing activity of responders has been reported to be significantly higher than that of non-responders [12], which precisely explains why we chose in the present study to discriminate neurons of interest on the basis of this same parameter (see also Discussion).

On average, 5-HT neuron impulse flow recovered up to 70% and 61% with respect to basal values, in the presence of fluvoxamine and citalopram respectively, after the administration of prucalopride (Figs. 1A and 1B). Statistically, the recovery appeared to be total in the case of fluvoxamine [within-design one-way ANOVA, F(2, 14) = 15.9, p<0.001; fluvoxamine alone vs basal p<0.001, fluvoxamine+prucalopride vs fluvoxamine alone p<0.05, fluvoxamine+prucalopride vs basal n.s. (Tukey's test); Fig. 1A], whereas it was only partial in the case of citalopram, in that the firing rate observed after prucalopride was still significantly lower than basal levels [within-design one-way ANOVA, F(2, 17) = 20.6, p<0.001; citalopram alone vs basal p<0.001, citalopram+prucalopride vs citalopram alone p<0.05, but citalopram+prucalopride vs basal p<0.05 (Tukey's test); Fig. 1B].

### Effect of acute co-administrations of citalopram and of the selective 5-HT_4_ agonist prucalopride on hippocampal pyramidal neuron activity

**Figure 2:**
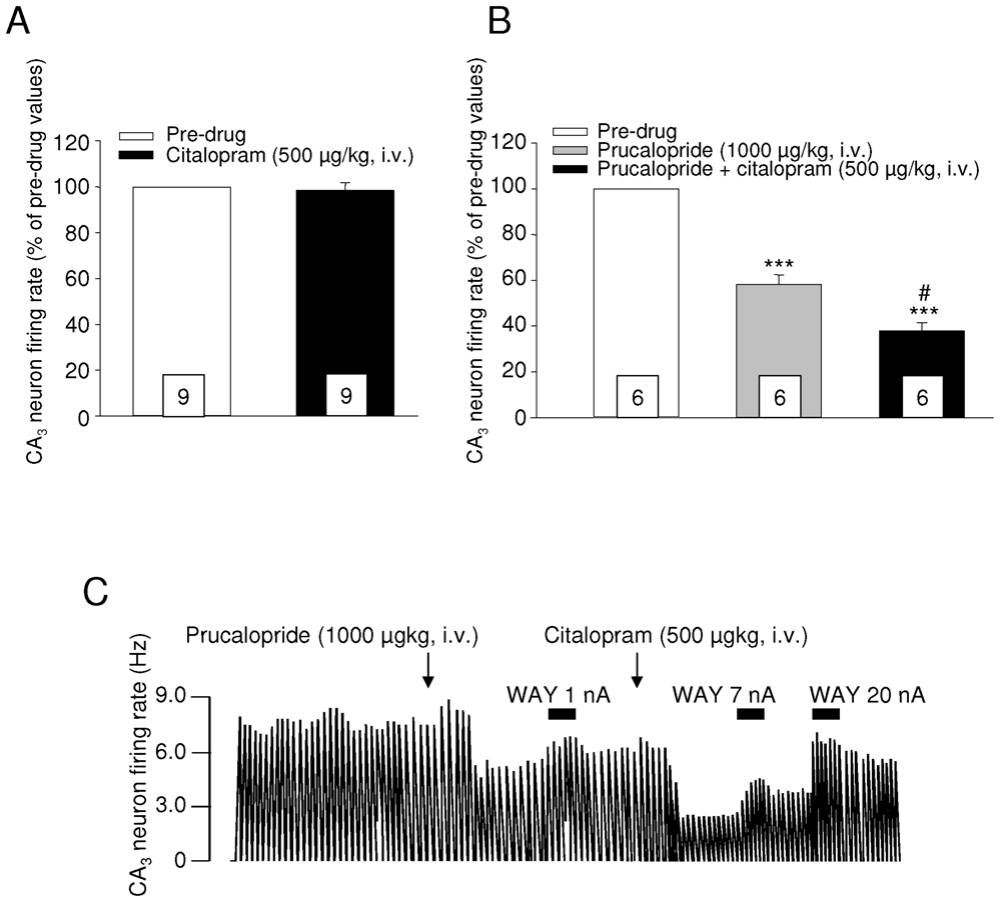
Effect of an acute, intravenous administration of citalopram (500 μg/kg), alone or subsequent to an i.v. dose of prucalopride (1000 μg/kg), on CA_3_ pyramidal neuron firing activity. **A** and **B**: Bar histograms represent the mean (± S.E.M.) percentage effect, in the absence or presence of prucalopride respectively, calculated for each neuron with respect to its basal firing rate (i.e. 100% level). The value at the bottom of each column represents the number of neurons tested (one single neuron recorded per rat). *** p<0.001 vs pre-drug, # p<0.05 vs prucalopride, Tukey's test. **C**: A typical example (integrated firing rate histogram) of the results summarized in **B**. The neuron chosen to illustrate this panel is also one of the two cells in which the effect of a local, microiontophoretic application of the selective 5-HT_1A_ antagonist WAY 100635 was tested (see Discussion for details).

It had previously been reported that in acute conditions, the dose of a given SSRI required to observe an inhibition of hippocampal pyramidal neurons is at least 5 to 10 times higher than that able to suppress the activity of DRN 5-HT neurons [21]. This difference is thought to be related to the fact that, at lower doses, 5-HT reuptake blockade concerns essentially the somatodendritic area, hence the suppression of firing, and that only a massive occupation of terminal sites produced by a high dose of the SSRI can elicit an elevation of extracellular 5-HT levels consistent enough to stimulate the inhibitory hippocampal 5-HT_1A_ postsynaptic receptors [21]–[22]. Based on this, we used citalopram at 500 µg/kg, i.v. This dose actually corresponds to no more than 3–4 times the ED50 we previously found for this drug, regarding its abolishing action on 5-HT neuron activity [14]. Not surprisingly therefore, and as shown in Fig. 2A, this treatment remained devoid of any effect in all the 9 neurons recorded. The picture, however, was different when citalopram was administered a few minutes after an intravenous dose of 1000 µg/kg of prucalopride. In well agreement with the data reported in our previous study [14], this treatment induced on its own a 42% reduction of pyramidal neuron activity in 6 of the 15 cells tested (Fig. 2B), whereas the remaining ones were not affected (not shown). And, in all neurons responding to prucalopride administration, the subsequent injection of citalopram further inhibited the firing frequency, which diminished by 62% with respect to basal values (Fig. 2B) [within-design one-way ANOVA, F(2, 17) = 169.3, p<0.001; prucalopride vs pre-drug and prucalopride+citalopram vs pre-drug p<0.001 each, prucalopride+citalopram vs prucalopride p<0.05, Tukey's test]. An example of this is shown in Fig. 2C, further illustrating the combined action of the two drugs in one single pyramidal neuron.

### Effect of chronic co-administrations of citalopram with a selective 5-HT_4_ receptor agonist on DRN 5-HT mean neuronal firing rate

**Figure 3:**
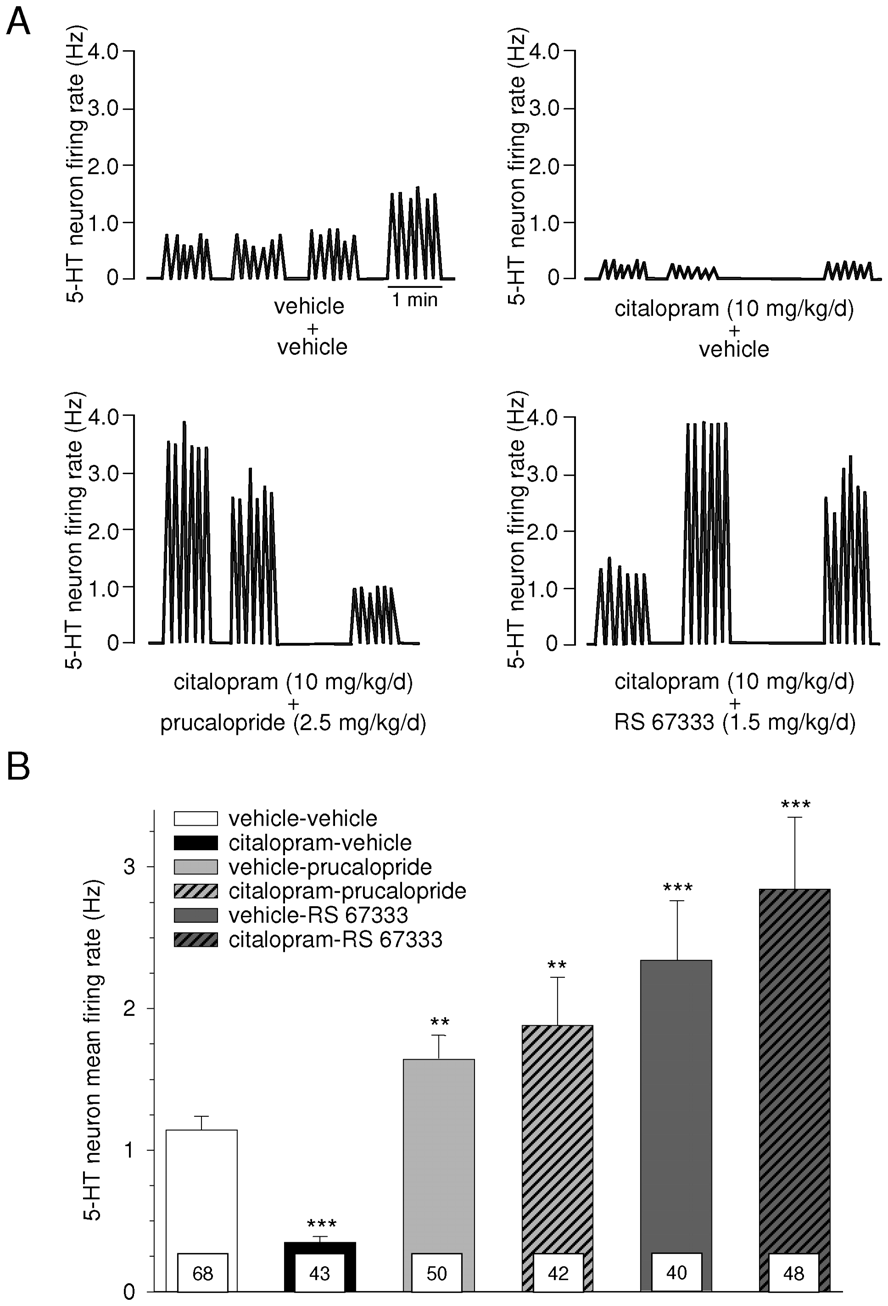
Effect of a 3-day co-treatment with citalopram, and either prucalopride or RS 67333, on the mean firing rate of DRN 5-HT neurons. In each rat, both citalopram (or its vehicle) and the 5-HT_4_ agonist (or its vehicle) were administered via osmotic minipumps inserted subcutaneously, and recordings were performed with the two minipumps still in place. **A**: Integrated firing rate histogram showing samples of DRN descents in different experimental groups; indicated doses refer to the total daily dosage. **B**: Summary of the results: bar histograms represent the mean (± S.E.M.) firing activity of 5-HT neurons, calculated on the basis of successive recording tracks performed along the DRN. Values at the bottom of each column indicates the total number of neurons recorded per group (vehicle-vehicle: n = 7 animals, citalopram-RS 67333: n = 5 animals, and n = 4 animals in the other groups). ** p<0.01 and *** p<0.001 vs vehicle/vehicle, Tukey's test.

In keeping with earlier results from our laboratory [13], [23], basal mean discharge of DRN 5-HT neurons in control (vehicle + vehicle) animals was found to be 1.14±0.1 Hz (Figs. 3A and 3B). Overall, the different chronic treatments (and combinations thereof) tested significantly modified this value [between-design one-way ANOVA, F(5, 290) = 9.6, p<0.001]. Thus, a 3-day continuous administration of citalopram (10 mg/kg/d) resulted in a dramatic decrease of 5-HT neuron activity, which dropped to 31% with respect to control levels (0.35±0.04 Hz) (Tukey's test, p<0.001 vs vehicle+vehicle; Fig. 3B). Virtually none of the 5-HT neurons recorded in the citalopram+vehicle group discharged above 0.5 Hz, whereas it was common to observe frequencies within the 1–1.7 Hz range in the vehicle+vehicle one (Fig. 3A). The number of spontaneously active 5-HT cells was also slightly, but significantly lower in citalopram-treated than in control rats (Table 1). By contrast, and in agreement with our previous report [13], both prucalopride (2.5 mg/kg/d) and RS 67333 (1.5 mg/kg/d) enhanced DRN 5-HT neuronal activity when they were continuously administered for 3 days. The results obtained herein are indeed very similar to those of the 2005 study, with mean values of 1.73±0.17 (+52%) and 2.16±0.32 Hz (+89%) for prucalopride and RS 67333, respectively (Tukey's test, p<0.01 and p<0.001 vs vehicle+vehicle, respectively; Fig. 3B). On their own, neither of the two 5-HT_4_ receptor agonists did affect the number of spontaneously active 5-HT cells per track (Table 1).

**Table 1:** Average number (± S.E.M.) of 5-HT neurons found per recording tracks (“descents”) performed along the DRN, in rats treated for three days with citalopram (cit, 10 mg/kg/d), prucalopride (pru, 2.5 mg/kg/d), RS 67333 (RS, 1.5 mg/kg/d) or their vehicle (veh).

	Treatment group					
	veh/veh	cit/veh	veh/pru	cit/pru	veh/RS	cit/RS
# of 5-HT neurons per track	4.54±0.5	2.75±0.7^a^	4.13±0.6	2±0.3^c^	4.1±0.8	1.95±0.3^c^

In each animal, two osmotic minipumps, filled with either a compound of interest or its vehicle, were inserted under the skin of the back under short-duration (<5 min) anaesthesia. Recordings were performed with the minipumps still in place. a, p<0.05; c, p<0.001 vs veh/veh (Student's *t*-test).

Most importantly, we found that a 3-day co-administration of the SSRI citalopram with a 5-HT_4_ receptor agonist resulted in an increase of DRN 5-HT neuron mean firing rate, displaying an amplitude similar (or even slightly superior) to that induced by each agonist alone. As illustrated in Fig. 3A, some of the 5-HT cells encountered along recording tracks displayed frequencies as high as 3.5 or even 4 Hz in both combination (citalopram + prucalopride and citalopram + RS 67333) groups. On average, the citalopram+prucalopride combined treatment enhanced 5-HT impulse flow by 65%, and the citalopram + RS 67333 one by 149% (Tukey's test, p<0.01 and p<0.001 vs vehicle + vehicle, respectively). Although this latter value appeared to be more consistent than the +89% effect elicited by RS 67333 alone, the two groups did not statistically differ from each other (Tukey's test, n.s.). However, both combined treatments significantly reduced the number of spontaneously active cells found per track, to about one-half with respect to control levels (Table 1).

### Effects of chronic co-administrations of citalopram and of a selective 5-HT_4_ receptor agonist on the reactivity of hippocampal pyramidal neurons to 5-HT_1A_ receptor blockade

The effects of chronic combined treatments were assessed on the responsiveness of hippocampal pyramidal neurons to systemic injection of the selective 5-HT_1A_ antagonist WAY 100635. For this purpose, the compounds of interest were delivered at the same dose regimen than above, and pyramidal neurons were recorded within the CA_3_ sub-field, before and after cumulative i.v. doses of WAY 100635. Since most pyramidal neurons are not spontaneously active under chloral hydrate anaesthesia, the use of a multi-barreled electrode coupled with microiontophoretic pumps was required, so that an ejection current of quisqualate permitted to maintain pyramidal neuron firing rate within the 3–6 Hz range. These experimental conditions have proved effective to reveal the manifestation of an inhibitory tonus mediated by hippocampal postsynaptic 5-HT_1A_ receptors [8], which constitutes so far a common and selective trait for all chronic antidepressant treatments [8], [24]–[25].

**Figure 4:**
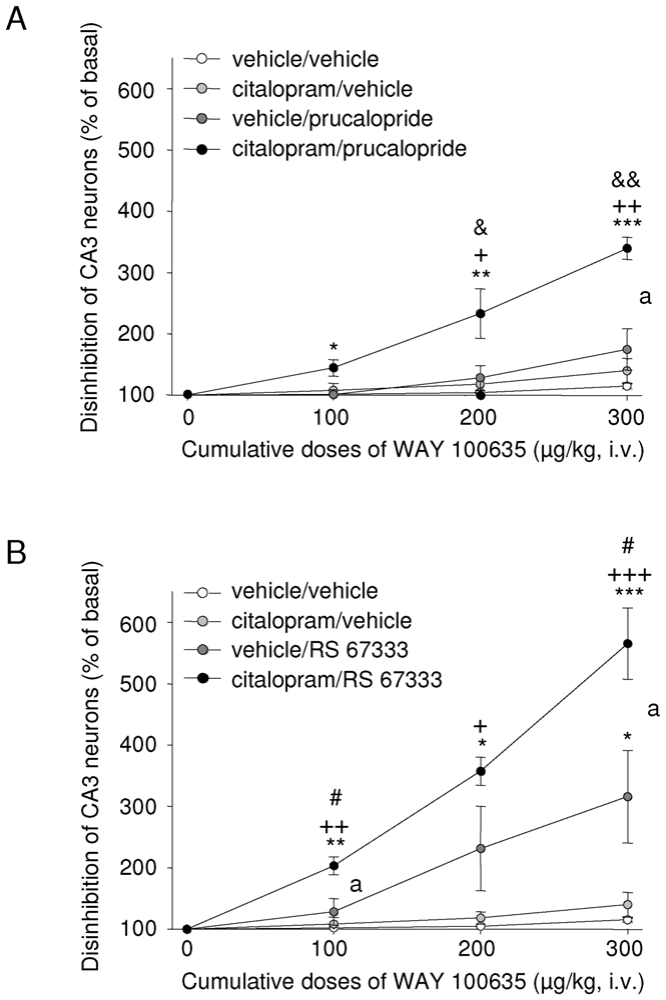
Effect of the combination of citalopram with a 5-HT_4_ agonist on the postsynaptic 5-HT_1A_ neurotransmission. Cumulative intravenous doses of the selective 5-HT_1A_ antagonist WAY 100635 were performed in rats continuously treated with **A**, the citalopram (10 mg/kg/d) + prucalopride (2.5 mg/kg/d) or **B**, the citalopram + RS 67333 (1.5 mg/kg/d) combination for three days, and their effects on the firing activity of hippocampal pyramidal neurons were recorded in the CA3 sub-field. Single-cell extracellular recordings were performed in chloral-hydrate anaesthetized animals, by using multiple-barrel glass microelectrodes combined with microiontophoretic pumps, and results expressed as the mean (± S.E.M.) percentage elevation of the firing rate with respect to pre-drug values (n = 4 animals in each group). All compounds (or their vehicle) were administered through the use of osmotic minipumps, inserted subcutaneously in the region of the back. Recordings were performed with the minipumps still in place. * p<0.05, ** p<0.01 and *** p<0.001 vs respective vehicle/vehicle values; + p<0.05, ++ p<0.01 and +++ p<0.001 vs respective citalopram/vehicle values; & p<0.05 and && p<0.01 vs respective vehicle/prucalopride values; # p<0.05 vs respective vehicle/RS 67333 values (Tukey's test). The “*a*” symbol indicates a p<0.05 significant interaction between the citalopram “pre-treatment” and the 5-HT_4_ agonist “treatment”, as revealed by the use of a two-way ANOVA.

We previously reported that after only three days of continuous administration, both prucalopride and RS 67333 are able to induce the apparition of such a tonus, at difference with citalopram, which remains inactive within this short time-frame [14]. As illustrated in Fig. 4, similar results were observed in the present study, with WAY 100635 displaying a dose-dependent excitatory effect in rats treated with the 5-HT_4_ agonists, and being devoid of any significant effect in both the vehicle/vehicle and citalopram/vehicle groups. In the presence of prucalopride alone, cumulative doses of 200 and 300 µg/kg increased the firing activity of pyramidal neurons to 129±20 and 175±34% of basal values, respectively (Fig. 4A); in the vehicle/RS 67333 group, the enhancement was more pronounced, reaching 128±22, 231±69 and 316±75% after 100, 200 and 300 µg/kg of WAY 100635, respectively (Fig. 4B). These results are remarkably close to those reported in our previous study [14]. The adjunction of citalopram spectacularly potentiated the effect of both 5-HT_4_ agonists. Indeed, in citalopram/prucalopride-treated animals, the intravenous administration of WAY 100635 (100, 200 and 300 m/kg) raised CA3 pyramidal neuron firing rate to 142±13, 233±40 and 340±18% with respect to control levels, respectively (Fig. 4A). In the citalopram/RS 67333 group, the enhancing action of WAY 100635 reached amplitudes as high as 203±14, 357±23 and 565±68% in the same conditions (Fig. 4B). The “synergetic” nature of these results was further confirmed by the use of two-way ANOVAs, which revealed the existence of significant interactions between the citalopram “pre-treatment” and the 5-HT_4_ agonists “treatment”, after the WAY 100635 doses of 300 µg/kg [pretreatment x treatment interaction, F(1, 15) = 7, p<0.05] (Fig. 4A), and 100 and 300 µg/kg [pretreatment x treatment interaction, F(1, 15) = 4.9, p<0.05, and F(1, 15) = 6.3, p<0.05] (Fig. 4B) in the presence of prucalopride and RS 67333, respectively. Also noteworthy was the finding that in most cases, and notably after the highest cumulative dose of WAY 100635, Tukey's tests (performed after significant between-designed one-way ANOVAs) indicated that combination groups were significantly different from both the citalopram/vehicle and the vehicle/5-HT_4_ agonists ones (see Figs. 4A and 4B).

### Effect of a 3-day combined treatment with the SSRI citalopram and the 5-HT_4_ receptor agonist RS 67333 on CREB phosphorylation in the hippocampus

**Figure 5:**
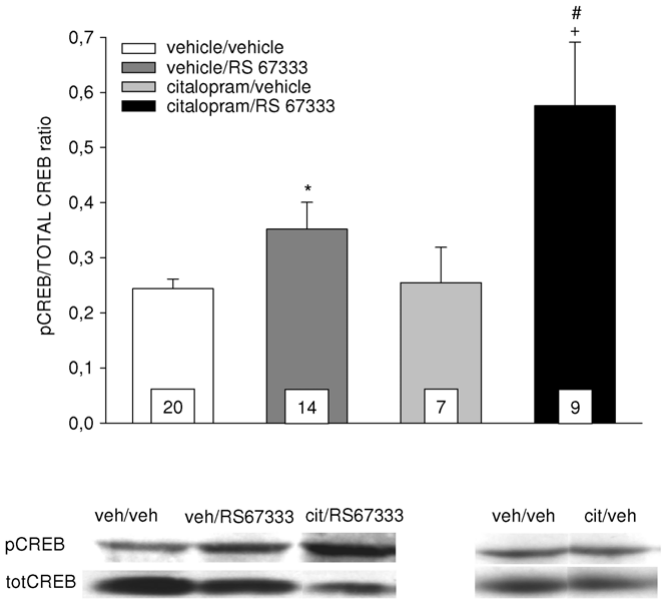
Effect of the continuous co-administration of citalopram (10 mg/kg/d) and RS 67333 (1.5 mg/kg/d) for 3 days on pCREB. The activation of CREB in hippocampal tissue was assessed by measuring phosphoCREB (pCREB) immunoreactivity. CREB phosphorylation was normalized according to the amount of protein present in each sample by expressing the data as a ratio of pCREB over total CREB immunoreactivity. Results represent mean ± SEM for the number of experiments indicated, for each treatment, by the value at the bottom of the column. Inset shows representative examples of pCREB immunoreactivity for different treatment conditions indicated on the histogram. All compounds were administered through the use of osmotic minipumps, inserted subcutaneously in the region of the back. * p<0,05 vs vehicle/vehicle, + p<0.05 vs citalopram/vehicle and # p<0.05 vs vehicle/RS 67333, Student's *t*-test.

As illustrated in Fig. 5, a statistical significance was found when all four groups were compared with a global ANOVA [between-design one-way ANOVA, F(3, 49) = 7.2, p<0.001]. Values in the citalopram/RS 67333 group were found to be significantly higher than in both the vehicle/vehicle and vehicle/RS 67333 ones (Tukey's test, p<0.001 and p<0.05, respectively), but surprisingly, in such statistical conditions the difference between vehicle/RS 67333 and vehicle/vehicle did not appear to be significant (Tukey's test, n.s.). However, when assessed with a Student's *t*-test, the difference was actually significant (p<0.05, Fig. 5), in agreement with a recent study from our laboratory showing that a 3-day treatment with RS 67333 alone was sufficient to increase the pCREB/CREB ratio within the hippocampus [14]. Indeed, and again, the ratio value found in the present study for the vehicle/RS 67333 group (0.35±0.05) is very close to what we obtained for the “RS 67333 group” in this previous work (0.37±0.02; [14]). Still, because present control ratios are on average slightly more elevated, the effect of RS 67333 appears to be less prominent (+44%, vs +105% in [14]), which is likely to explain why the Tukey's test did not reveal a statistical significance after a global ANOVA.

In the presence of citalopram, RS 67333 was still able to augment phosphorylation of hippocampal CREB (citalopram/RS 67333 vs citalopram/vehicle p<0.05, Student's *t*-test; Fig. 5). More interestingly, the facilitatory action of RS 67333 was actually significantly potentiated by the SSRI. Thus, citalopram “pre-treatment” significantly interacted with RS 67333 “treatment”, as demonstrated by the use of a two-way ANOVA [pre-treatment x treatment interaction, F(1, 49) = 3.8, p<0.05]. Citalopram, which, as already shown [14], remained without effect on its own after 3 days of administration (citalopram/vehicle vs vehicle/vehicle, n.s., Student's *t*-test), exerts therefore a synergetic action on the rapid facilitatory effect of RS 67333. This conclusion is supported by the significant result of the Student's *t*-test used to compare the two groups of animals treated with RS 67333 (citalopram/RS 67333 vs vehicle/RS 67333, p<0.05; Fig. 5), which also confirmed the Tukey's test mentioned above.

### Effects of co-administrations of the 5-HT_4_ agonist RS 67333 with various SSRIs in the FST

**Figure 6:**
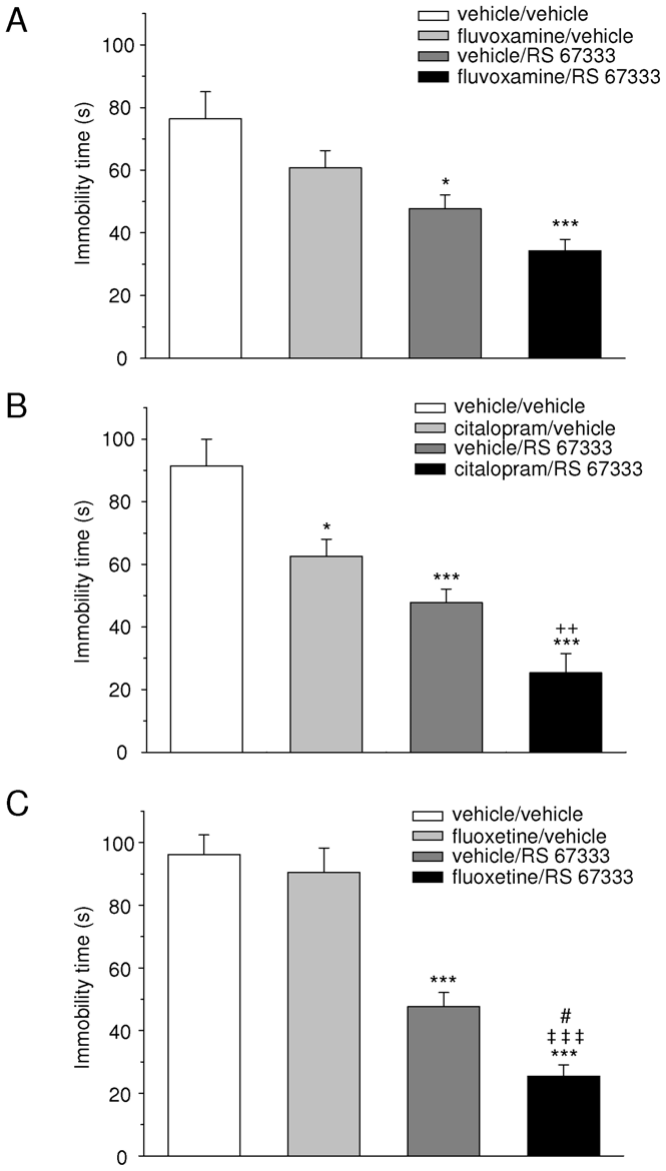
Effect of RS 67333 (1.5 mg/kg, i.p.), in combination with various SSRIs, on the time spent immobile in the FST. The SSRIs used were **A** fluvoxamine (10 mg/kg, i.p.), **B** citalopram (10 mg/kg, i.p.), and **C** fluoxetine (10 mg/kg, i.p.). All data are expressed as mean ± S.E.M. of eight animals per group (except for the vehicle/RS 67333 group which comprises 12 animals), and are from an observation of 4 min duration. Rats experienced a pre-test session (15 min) 24 h before the test session. In each animal, RS 67333 (or its vehicle) and the SSRI (or its vehicle) were administered almost concomitantly (within a 30 s interval), 30 min before the test session; the sites chosen for i.p. injections were always symmetrically opposed with respect to the abdomen midline. * p<0.05 and *** p<0.001 vs vehicle/vehicle, ++ p<0.01 vs citalopram/vehicle, ‡‡‡ p<0.001 vs fluoxetine/vehicle and # p<0.05 vs vehicle/RS 67333, Tukey's test.

As shown in Fig. 6, the effect of RS 67333 on time of immobility was, to the lesser, additive with that of fluvoxamine, citalopram and fluoxetine. More specifically, the between-design one-way ANOVA was significant in all three experiments [Fig. 6A, F(3, 33) = 7.5; Fig. 6B, F(3, 33) = 18.9; Fig. 6C, F(3, 33) = 34.9; p<0.001 for all]. The effect of fluvoxamine alone (−21%) failed to reach statistical significance (Tukey's test, n.s.), at difference with that of RS 67333 (−38% with respect to this control group, p<0.05, Tukey's test) (Fig. 6A). When combined together, these compounds reduced time of immobility by 55% with respect to the vehicle/vehicle group (p<0.001, Tukey's test, Fig. 6A); this value is, obviously, very close to 59%, which would theoretically correspond to the sum of individual influences of each drug. Citalopram induced on its own a significant reduction of time of immobility (−32%, p<0.05 vs vehicle/vehicle, Tukey's test, Fig. 6B). In this experiment, the effect of RS 67333 appeared slightly more pronounced (−48%, p<0.001 vs vehicle/vehicle, Tukey's test, Fig. 6B), because the control group displayed values somewhat higher than those of Fig. 6A (91.4±8.6 s vs 76.4±8.7 s). However, in this case also, the co-administration of RS 67333 with the SSRI resulted in an effect (−72%, p<0.001 vs vehicle/vehicle, Tukey's test, Fig. 6B), which amplitude was almost similar to the sum of the two other ones. Interestingly, the citalopram/RS 67333 and citalopram/vehicle groups were statistically different from each other (p<0.01, Tukey's test, Fig. 6B), and that was also almost the case concerning citalopram/RS 67333- and vehicle/RS 67333-treated animals (p = 0.09, Tukey's test). The administration of fluoxetine had virtually no effect on the time spent immobile (−6%, n.s. vs vehicle/vehicle, Tukey's test, Fig. 6C). On the other hand, and similarly to the above results, RS 67333 induced a 50% reduction of this parameter (p<0.001 vs vehicle/vehicle, Tukey's test, Fig. 6C). The concomitant administration of fluoxetine and RS 67333 strongly attenuated time of immobility (−74%, p<0.001 vs vehicle/vehicle, Tukey's test, Fig. 6C). On the basis of these latter data, it could have been suggested that RS 67333 and fluoxetine actually exerted a “synergetic” action, namely that the drugs potentiated the action of each other rather than simply having added their potency. To verify this possibility, we performed a two-way ANOVA, with fluoxetine as the “pre-treatment” factor, and RS 67333 as the “treatment” factor. The statistical assessment did not reveal a significant interaction [two-way ANOVA, pre-treatment x treatment interaction, F(1, 35) = 2, n.s.], ruling out the possibility of a true “synergy” between fluoxetine and RS 67333 in the FST. However, it remains that this time, the fluoxetine/RS 67333 group was not only statistically different from the fluoxetine/vehicle one, but also reached significance when compared to vehicle/RS 67333-treated animals (p<0.001 each, Tukey's test, Fig. 6C).

## Discussion

The present study shows that the adjunction of an SSRI to a 5-HT_4_ agonist strongly potentiates the antidepressant-like properties of the latter in several electrophysiological, molecular and behavioral paradigms. Moreover, this combined treatment displays the same rapidity of action than that observed with the agonist alone, therefore appearing to constitute a very promising strategy in a clinical perspective.

As previously reported [18]–[20], an acute administration of the SSRIs fluvoxamine and citalopram almost suppressed the activity of all DRN 5-HT neurons recorded. Because 5-HT_4_ receptor agonists have been reported to increase the firing rate of a subpopulation (47%) of “responding” 5-HT neurons [12], we tested the ability of a subsequent injection of the selective 5-HT_4_ agonist prucalopride [26] to reverse the SSRI-induced inhibition. Responding neurons display a significantly higher average basal firing rate than that of non-responders [12], and thus, to optimize the discrimination between the two populations, the 5-HT neurons selected for recordings had been chosen for discharging within either the lower (≤1 Hz) or the upper (≥2 Hz) range of usual frequencies. We found that prucalopride counteracted the inhibition induced by both SSRIs in all “upper-frequency” cells, whereas it remained without any effect in the “lower-frequency” ones. These results confirm, once again [12], that a high basal firing rate constitutes a good predictor concerning the 5-HT_4_-responding nature of a DRN 5-HT neuron. More importantly, they also strongly suggest that the mechanisms underlying, respectively the 5-HT_4_-dependent excitatory control and the SSRI-induced decrease of 5-HT function, are fully independent from each other. Indeed, it is now well established that the latter results from an increased stimulation of 5-HT_1A_ inhibitory autoreceptors, which itself is consecutive to the strong elevation of extracellular 5-HT levels that follows 5-HT reuptake blockade in the somatodendritic area [17]–[18], [20]. It has also been reported that the firing activity of almost one-half of DRN 5-HT neurons can be reduced by stimulating some postsynaptic 5-HT_1A_ receptors, localized within the medial prefrontal cortex (mPFC) and that trigger a negative “long-loop feedback” [17], [27]. However, this regulatory mechanism does not participate in the suppressing action of SSRIs, the enhancement of 5-HT outflow elicited by these drugs being apparently not consistent enough to permit the stimulation of cortical 5-HT_1A_ receptors [17].

In contrast, the excitatory control exerted by 5-HT_4_ receptors on 5-HT neuronal impulse flow does not seem to involve any presynaptic modulation [13], and has been suggested to originate from the mPFC through the activation of a positive “long-loop feedback” [13]. The fact that neither fluvoxamine nor citalopram could preclude the facilitatory action of prucalopride in responding neurons suggests, therefore, that the elevation of 5-HT extracellular levels induced by an SSRI is also not sufficient for stimulating cortical 5-HT_4_ receptors. Interestingly, calculating the difference between the percentages found in the SSRI+prucalopride condition (70% and 61% with respect to basal levels for fluvoxamine and citalopram, respectively) and in the SSRI alone one (26% and 18%, respectively) leads to a quasi-constant value (44% vs 43%), regardless of the SSRI considered. That the effect of prucalopride remains so remarkably stable in the presence of two different, structurally unrelated compounds with differential potencies as 5-HT reuptake blockers [28] further supports the idea that the 5-HT_4_-mediated control on 5-HT neurons is independent from SSRI-triggered mechanism(s) of action. Finally, the facilitatory action of 5-HT_4_ receptor stimulation on 5-HT impulse flow permitted to unmask the effectiveness of a low dose of citalopram on postsynaptic targets. The i.v. injection of 500 µg/kg of citalopram, devoid of any effect on its own, significantly reduced CA_3_ pyramidal neuron activity when administered after prucalopride. Usually, such an effect is only observed in the presence of high doses of SSRIs [21]–[22], because the elevation of 5-HT outflow induced by these compounds is a passive phenomenon, strongly depending on electrical activity [21]. When this parameter is almost null, a massive occupation of reuptake sites is therefore required to increase 5-HT transmission at the postsynaptic level [21]–[22]. Our results confirm that this is no more required if 5-HT neuron firing rate becomes “normalized”, as it is the case in the presence of a 5-HT_4_ agonist. The postsynaptic 5-HT receptor(s) responsible for this inhibition of pyramidal neuron firing remains to be characterized, but it is likely that the 5-HT_1A_ type contributes, at least partially, to its expression. The effect of a local, microiontophoretic application of WAY 100635 was tested in two of the 6 responding neurons, and in both cases, it was actually able to reverse the effect of the prucalopride+citalopram combination (Fig. 2C).

The picture was slightly different when citalopram was co-administered continuously for three days with either prucalopride or RS 67333, an other selective 5-HT_4_ agonist [29]. In this case, indeed, the concomitant stimulation of 5-HT_4_ receptors did not only counteract the suppressing action of the SSRI, but actually resulted in an increase of the average DRN 5-HT neuron firing frequency, which amplitude was comparable to that induced by each 5-HT_4_ agonist alone. In our opinion, this finding is likely to be related to desensitization processes occurring at the level of somatodendritic 5-HT_1A_ autoreceptors, as the analysis of the number of spontaneously neurons found per track in the different experimental groups also indirectly suggests. Thus, in agreement with earlier studies [30], a 3-day treatment with citalopram alone strongly reduced (−70%) the mean activity of central 5-HT neurons. It is known that, after such a short duration of treatment, 5-HT_1A_ autoreceptors are still fully functional [9], [30]. Consequently, the presence of citalopram results in either a marked lowering of 5-HT impulse flow, hence the average reduction mentioned above, or in its total suppression, hence the slight reduction in the number of active cells found per descent (Table 1) [9], [30]. On the other hand, the continuous administration of a 5-HT_4_ agonist for three days elicits a desensitization of 5-HT_1A_ autoreceptors, which concerns apparently both “responding” and non-responding 5-HT neurons [14]. This is only a partially achieved process, however, as a stronger activation of autoreceptors can still suppress 5-HT neuronal firing rate at this point [14]. Assuming that the summed influences of an SSRI and of a 5-HT_4_ agonist should result in a massive elevation and diffusion of extracellular 5-HT within the DRN, this property could account for the 50% reduction of spontaneously firing cells, observed in both combined treatment groups (Table 1). Silent neurons would correspond to the (about) 50% of non-responders, in which by definition 5-HT_4_ agonism can not compensate for an activation of 5-HT_1A_ autoreceptors. In the case of responders, it is possible that the elevation of 5-HT levels is so important in the immediate vicinity of the somatodendritic region, that their autoreceptors are this time not partially, but already fully desensitized at day 3 after the onset of treatment. This would explain the finding that co-administrations have similar effects than 5-HT_4_ agonists alone, and do not simply permit a recovery of 5-HT activity as observed in acute conditions.

Additional experiments are obviously required to confirm the above hypotheses, but it it is clear that, whatever the underlying mechanisms, the facilitatory effect of a 3-day treatment with a 5-HT_4_ agonist on the average 5-HT impulse flow is not altered by the concomitant adjunction of an SSRI. However, because only one-half of 5-HT neurons are active in such conditions, the question remains whether these promising “presynatpic” results can effectively translate into an increased efficacy at the postsynaptic level. To address this point, we assessed the influence of a 3-day combined treatment, on the response of hippocampal pyramidal neurons to an acute injection of the selective 5-HT_1A_ antagonist WAY 100635. As previously shown [14], [23]–[25], this experimental protocol allows unveiling the manifestation of a 5-HT_1A_-mediated inhibitory tonus within the hippocampus, a specific feature induced by all currently used antidepressant treatments [24]. Indeed, WAY 100635 is virtually devoid of effect on DRN 5-HT activity in anaesthetized rats [for review, 31], and any change observed in such conditions is therefore very likely to reflect postsynaptic events. In agreement with earlier studies [14], our results indicate that an increased 5-HT_1A_ tone is already observable after three days of treatment with either prucalopride or RS 67333. They also show that the concomitant presence of citalopram does not preclude this effect of 5-HT_4_ receptor agonists. This should be regarded as a critical point, the rapidity of action of the latter in the same experimental paradigm having constituted one of the rational bases to propose their use as fast-acting antidepressants [14]. More importantly even, the response to WAY 100635 was much more pronounced in the case of co-administrations, indicating the presence of a more consistent 5-HT tonus. Thus, despite the 50% reduction of spontaneously active 5-HT neurons observed in these conditions, it appears that the raphé-hippocampus transmission was globally optimized. Statistical analysis of the experiments, using two-way ANOVAs, revealed that SSRI “pretreatment” interacted significantly with 5-HT_4_ agonists “treatments”, confirming the “synergetic” (i.e more than additive) nature of the combination. At difference with acute experiments, in this case the firing activity of 5-HT neurons was not normalized, but actually facilitated by the presence of 5-HT_4_ agonists. Considering the above discussed relationship between efficacy of reuptake blockade and impulse-flow, it is therefore not surprising that the SSRI displayed a more consistent influence at the terminal level, and that its effect was not simply added to that of the agonist. Interestingly, together these results also indirectly suggest that the efficacy of the combined treatment might improve over time. Thus, if an incomplete desensitization of 5-HT_1A_ autoreceptors is actually responsible for the silencing of 50% of 5-HT neurons at day 3 (see above), it is reasonable to expect a recovery after 2-3 weeks, as observed when citalopram is given alone [9], [30]. The resulting increase of 5-HT transmission would then be likely to “sum up” with that already triggered by the combined treatment, and related to “5-HT_4_-responding” 5-HT neurons. Indeed, the excitatory effect induced by 5-HT_4_ agonists displays a similar amplitude after 3 or 21 days of continuous administration [13]. Long-term (3 weeks) experiments are currently underway to confirm this possibility.

That the two classes of compounds have a synergetic action when chronically administered for three days was confirmed by assessing their combined influence on CREB activation. We recently reported that 5-HT_4_ agonists are already able to increase the hippocampal pCREB/CREB ratio within such a short time-frame [14], a property which, again, is typical of classical antidepressants but requires a prolonged (2–3 weeks) treatment with these molecules to manifest [32]–[34]. In the present study, similarly to what was observed on the 5-HT_1A_-mediated tonus in electrophysiological experiments, citalopram had no effect on its own, but strongly enhanced the efficacy of RS 67333. This remarkable parallelism of results between the two experimental paradigms further supports the idea of a direct relationship linking the raphé-hippocampus 5-HT transmission and the mechanisms triggering CREB activation [35]–[36]. It also indirectly suggests that the effect of 5-HT_4_ agonists on pCREB formation might not result from a dual action, namely a direct stimulation of the 5-HT_4_ receptors located on pyramidal neurons in addition to a global facilitation of 5-HT function, as previously proposed [37]. Indeed, if it had been the case in the present experimental conditions, citalopram should have competed with RS 67333 to stimulate hippocampal 5-HT_4_ receptors, and consequently to induce the phosphorylation of CREB. The occurrence of a synergetic action would have been therefore mostly improbable. Again, this hypothesis remains to be experimentally confirmed, and lesion/depletion studies are currently underway in our laboratory to determine the extent to which hippocampal 5-HT_4_ receptors are involved in the facilitatory effect of 5-HT_4_ agonists on CREB activation.

Altogether, the above results indicate that a combined SSRI/5-HT_4_ agonist treatment displays the same rapidity of action than the agonist alone to induce electrophysiological and molecular markers, specific of an antidepressant action. The adjunction of the SSRI, however, results in a dramatic potentiation of the markers amplitude, suggesting an even more promising clinical perspective. “Augmentation” strategies, aimed at helping conventional antidepressants to sustain their actions within the brain, appear actually to constitute an effective option for the two-thirds of patients who do not respond (or only partly do) to their treatment [16]. The improved antidepressant potential of the combination was further confirmed by the results found in FST experiments. Although this behavioral test can be performed in acute conditions, and does not reflect the need for prolonged treatment, it remains highly reliable to predict the ability of a given compound to reveal effective as an antidepressant [38]. Our results clearly show that the effect of RS 67333 was potentiated by a number of SSRIs, to the lesser in an additive manner, or almost synergistically in the case of fluoxetine. Again, this pattern was strikingly similar to what was observed in electrophysiological and molecular paradigms, strengthening the idea that central 5-HT neurotransmission plays also a pivotal role in antidepressant-induced behavioral changes [14]–[15], [36]. Obviously, such impressive effects induced by the SSRI/5-HT_4_ agonist association on the 5-HT transmission-pCREB-behavior “continuum” strongly support its use as a bi-therapy strategy for the treatment of depression. As recently pinpointed [37], there is still a need for safe 5-HT_4_ agonists able to enter the brain, and acceptable for clinical trials. Provided this critical step could be achieved however, the amount of SSRIs already available warranties the possibility to test multiple combinations, aimed at optimizing therapeutic outputs. For instance, the fact that a low dose of citalopram is already effective on postsynaptic targets when co-administered with a 5-HT_4_ agonist (Fig. 2) opens the perspective of reducing the doses of SSRI administered, in order to reduce side-effects as well. It is also of importance to mention that only one dose (1.5 mg/kg) of RS 67333 has been tested in the present study. It was chosen on the basis of previous reports, showing robust and consistent influence on the targeted parameters of interest (5-HT neuron firing rate, CREB phosphorylation) [13]–[14]. The fact that the amplitudes of effect were even higher when the same dose of compound was combined with SSRIs clearly indicates that, in this case, the aimed “augmentation” was actually effective. We did not, however, perform a complete dose-response study to determine which dose(s) of the 5-HT_4_ agonist permit(s) to observe an optimal augmentation. Obviously, such preclinical assessments will become required once safe, brain penetrant 5-HT_4_ agonists will have been designed to conduct clinical trials.

This would constitute one of the first attempts to use a bi-therapy approach as a first-line solution to treat depression. About 15 years ago, after a series of cleverly conducted preclinical investigations, the group led by F. Artigas started to perform clinical tests also based on a dual administration strategy [39]. The idea consisted of giving the 5-HT_1A_ receptor blocker pindolol concomitantly with a conventional antidepressant; because experimental data had suggested that pindolol might act preferentially on 5-HT_1A_ autoreceptors, it was thought that such a combination could permit to reduce the delay of action of the treatment (see Introduction) [39]–[40]. Unfortunately, it appears that in humans, pindolol is also able to block significantly postsynaptic 5-HT_1A_ receptors, especially at the higher doses used [41]–[42]. Considering the crucial role played by these receptors in the effect of antidepressants [10], [15], it is therefore not surprising that the different trials conducted led to controversial and variable results, with no consistent improvement observed concerning the amplitude of the therapeutic efficacy, and a reduced delay that was not systemically found [40]. On the other hand, the present results suggest that the mechanisms of action underlying the potential antidepressant properties of 5-HT_4_ receptor agonists and those of SSRIs do not interfere with each other. It is therefore reasonable to hope that their combination may constitute in the future one of the routine bi-therapies used in depressed patients.

## Materials and Methods

### Animals

Experiments were carried out in male Sprague-Dawley (Charles River, St-Constant, Québec, Canada, and Harlan, Gannat, France) rats, weighing 250–300 g and kept under standard laboratory conditions (12∶12 light-dark cycle with free access to food and water, light starting from 7 AM). Experiments were performed in conformity with the Canadian Guide to the Care and Use of Experimental Animals (Vol. 1, 2nd edition, 1993) and with the guidelines of the French Ministry of Agriculture (87/847, modified May, 2001). All efforts were made to minimize the number of animals used in each experimental group.

### Drugs and chemicals

The following compounds were used: prucalopride monohydrochloride, fluvoxamine maleate, citalopram hydrobromide, fluoxetine hydrochloride (gifts from Janssen, Solvay, Lundbeck and Eli Lilly laboratories, respectively), RS 67333 hydrochloride (Tocris Cookson Inc., Ellisville, MO, USA), WAY 100635 hydrochloride (Research Biochemicals, Natick, MA, USA). All compounds were diluted in distilled water, and in each case, drug dosage refers to the free base. All other reagents were the purest commercially available. For chronic treatments, prucalopride (2.5 mg/kg/day), RS 67333 (1.5 mg/kg/day), citalopram (10 mg/kg/day) or the vehicle were delivered through osmotic minipumps (Alza, Palo Alto, CA, USA), inserted subcutaneously in the region of the back under short-duration (≤5 min) halothane anaesthesia. Given that these experiments were performed to assess the effect of the concomitant administration of an SSRI with a 5-HT_4_ receptor agonist, in each animal two minipumps, containing either the compound of interest or its vehicle, were simultaneously inserted.

### Extracellular recordings of DRN 5-HT neurons

Recordings were performed using single-barreled glass micropipettes. Electrodes were filled with a 2 M NaCl solution saturated with Fast Green FCF, resulting in an impedance of 2–5 MΩ. Rats were anaesthetized with chloral hydrate (400 mg/kg, i.p.) and placed in a stereotaxic frame. A burr hole was drilled on the midline 1 mm anterior to lambda. DRN 5-HT neurons were encountered over a distance of 1 mm starting immediately below the ventral border of the Sylvius aqueduct. These neurons were identified using the classical criteria: a slow (0.5–2.5 Hz) and regular firing rate and long-duration (0.8–1.2 ms) positive action potentials [43]. At the end of the experiments, a 25 µA cathodal current was passed through the recording electrode to leave a Fast Green deposit at the recording site. Animals were sacrificed with an i.v. overdose of chloral hydrate, and the brain removed. The site of recording was verified under microscope immediately after experiments. In the case of chronic administrations, recordings were performed with the two minipumps still in place.

### Extracellular recordings from hippocampal CA_3_ pyramidal neurons and microiontophoresis

Recording and microiontophoresis were performed with five-barreled glass micropipettes broken back to 8–12 µm under microscope control. The central barrel was filled with the same solution as in the DRN and was used for extracellular unitary recordings. Pyramidal neurons were identified by their large amplitude (0.5–1.2 mV) and long-duration (0.8–1.2 ms) simple spikes alternating with complex spike discharges [44]. The side barrels contained the following solutions: quisqualate (1.5 mM in 200 mM NaCl, pH 8), WAY 100635 (15 mM in 200 mM NaCl, pH 4), and 2 M NaCl used for automatic current balancing. Rats were mounted in the stereotaxic apparatus and the micropipettes were lowered at 4.2 mm lateral and 4.2 anterior to lambda into the CA_3_ sub-region of the dorsal hippocampus. In the case of chronic administrations, recordings were performed with the two minipumps still in place.

### Assessment of CREB and pCREB immunoreactivities

Following decapitation (performed under halothane anaesthesia) rat brains were dissected on ice cold artificial cerebrospinal fluid (125 mM NaCl, 2.4 mM KCl, 0.83 mM MgCl_2_, 1.1 mM CaCl_2_, 0.5 mM KH_2_PO_4_, 0.5 mM NaSO_4_, 27 mM NaHCO_3_, 10 mM glucose, 10 mM Hepes, pH 7.4). Isolated hippocampi (1–2 mg wet tissue/100 µl) were homogenized in solubilization buffer containing 20 mM Hepes pH 7.9, 0.4 M NaCl, 20% (v/v) glycerol, 1% (v/v) Nonidet P-40, 5 mM MgCl_2_, 0.5 mM EDTA, 0.1 mM EGTA, 1 mM phenylmethanesulfonyl fluoride, 1 µM okadaic acid, 5 mM dithiothreitol, 5 µg/ml leupeptin, 5 µg/ml soybean trypsin inhibitor, and 10 µg/ml benzamidine by means of a dounce homogenator. Homogenates were then adjusted to a concentration of 2 mg protein/ml and incubated on ice for 30 min, after which they were centrifuged for additional 30 min at 15,000 g. The supernatant was discarded and SDS sample buffer was added to the pellet for posterior immunoblot analysis. For detection of CREB activation samples were sonicated and then boiled for 5 min before loading for SDS-PAGE that was performed as previously described [45] using a 4% stacking gel and 10% separating gel. Proteins resolved in SDS-PAGE were then transferred from gels onto nitrocellulose (50 mA, 16 h, Bio-Rad Mini-Trans Blot apparatus) and pCREB detected by probing membranes with anti-pCREB monoclonal antibody (1B6) from Cell Signaling Technology (1∶1000). Total CREB contents was determined after stripping by using 1∶1000 dilution of anti-CREB antibody (Cell Signaling Technology). Secondary antimouse (1∶5000; Sigma) or antirabbit (1∶40000; Amersham) horseradish-conjugated antibodies and enhanced chemiluminescence detection reagents (NEN Life Science Products) were used to reveal blotted proteins. Relative intensities of the labeled bands were analyzed by densitometric scanning using MCID (Imaging Research Inc) and CREB-activation was expressed as the ratio between pCREB and total CREB present in each sample.

### Forced swimming test

The FST was performed by using a two-sessions procedure modification of the protocol originally described by Porsolt et al. [46]. Briefly, rats experienced a pre-test session followed 24 hours later by a test session. For both the pre-test and the test sessions, conducted under low illumination (15 W), the animals were placed in a plastic cylindrical tank (50 cm high by 20 cm in diameter) filled with water at 24°±1°C, with a depth of 40 cm, for which the hind limbs could not reach the tank floor. In all experiments, the pre-test was carried out for 15 min and the test for 5 min in the same tank but the last 4 min only were analyzed. RS 67333 (1.5 mg/kg, i.p.) or its vehicle was administered concomitantly with either fluvoxamine (10 mg/kg, i.p.), citalopram (10 mg/kg, i.p.), fluoxetine (10 mg/kg, i.p.) or their vehicle 30 min before the test session. All experiments were carried out within a single step (i.e. only one pre-test and one test session have been conducted). Overall, ten (10) experimental groups have been performed: vehicle + RS 67333, three [SSRI + vehicle], three [SSRI + RS 67333] and three [vehicle + vehicle] (control) groups. Following both pre-test and test sessions, rats were dried with a towel and kept warm for 30 min before returning in their home cage. A camera coupled with a computer recorded on line animal behavior during the FST through a specialized digital interface (Videotrack, ViewPoint, Lyon, France). This interface underscored on line the subtraction of video frames. Immobility time in FST was derived from the number of frames (every 40 ms) being below a predefined threshold over FST duration. This threshold was preliminarily set up in order to obtain about 95% of the corresponding frames classified as immobile for a non-swimming rat in its water tank.

### Statistical analysis

All data are expressed as means ± SEM unless otherwise specified. The effects of acute administration of prucalopride, either on the fluvoxamine- and citalopram-induced inhibition of 5-HT neuron activity, or on hippocampal neuron firing rate, were assessed using a within-design one-way ANOVA, followed by Tukey's *post hoc* test. The difference between the number of spontaneously active DRN 5-HT neurons found per recording track in control vs treated animals was evaluated using the Student's *t*-test. A Student's *t*-test was also performed to assess the effect of RS 67333 on hippocampal CREB phosphorylation, either in the absence or in the presence of citalopram, as well as to compare the vehicle/RS 67333 group with the citalopram/RS 67333 one (see Results for more details). In other experiments, differences between groups were assessed using between-design one-way ANOVAs, followed by *post-hoc* analysis (Tukey's test) when multiple comparisons were necessary. In some cases, two-way ANOVAs were also used to determine whether pretreatment (SSRI) interacted with treatment (5-HT_4_ agonist).
